# Metabolic engineering of a stable haploid strain derived from lignocellulosic inhibitor tolerant *Saccharomyces cerevisiae* natural isolate YB-2625

**DOI:** 10.1186/s13068-023-02442-9

**Published:** 2023-12-06

**Authors:** Ronald E. Hector, Jeffrey A. Mertens, Nancy N. Nichols

**Affiliations:** Agricultural Research Service, USDA, National Center for Agricultural Utilization Research, (Bioenergy Research), 1815 N University, Peoria, IL 61604 USA

**Keywords:** Xylose, Lignocellulosic inhibitors, Inhibitor-tolerant yeast, Renewable fuels

## Abstract

**Background:**

Significant genetic diversity exists across *Saccharomyces* strains. Natural isolates and domesticated brewery and industrial strains are typically more robust than laboratory strains when challenged with inhibitory lignocellulosic hydrolysates. These strains also contain genes that are not present in lab strains and likely contribute to their superior inhibitor tolerance. However, many of these strains have poor sporulation efficiencies and low spore viability making subsequent gene analysis, further metabolic engineering, and genomic analyses of the strains challenging. This work aimed to develop an inhibitor tolerant haploid with stable mating type from *S. cerevisiae* YB-2625, which was originally isolated from bagasse.

**Results:**

Haploid spores isolated from four tetrads from strain YB-2625 were tested for tolerance to furfural and HMF. Due to natural mutations present in the HO-endonuclease, all haploid strains maintained a stable mating type. One of the haploids, YRH1946, did not flocculate and showed enhanced tolerance to furfural and HMF. The tolerant haploid strain was further engineered for xylose fermentation by integration of the genes for xylose metabolism at two separate genomic locations (*ho*∆ and *pho13*∆). In fermentations supplemented with inhibitors from acid hydrolyzed corn stover, the engineered haploid strain derived from YB-2625 was able to ferment all of the glucose and 19% of the xylose, whereas the engineered lab strains performed poorly in fermentations.

**Conclusions:**

Understanding the molecular mechanisms of inhibitor tolerance will aid in developing strains with improved growth and fermentation performance using biomass-derived sugars. The inhibitor tolerant, xylose fermenting, haploid strain described in this work has potential to serve as a platform strain for identifying pathways required for inhibitor tolerance, and for metabolic engineering to produce fuels and chemicals from undiluted lignocellulosic hydrolysates.

**Supplementary Information:**

The online version contains supplementary material available at 10.1186/s13068-023-02442-9.

## Background

To generate biomass-derived sugars for producing renewable fuels and chemicals using microbial fermentation, pretreatment of biomass is required. Common pretreatment approaches utilize steam explosion and dilute acid (reviewed in [1]). The resistance of biomass necessitates the use of harsh conditions and leads to the production of an array of compounds (e.g., furan aldehydes, aliphatic acids, and phenolic compounds) that inhibit growth and fermentation (reviewed in [2, 3]). Because the presence of inhibitors is a barrier to efficient use of lignocellulosic hydrolysates, multiple approaches to removing or detoxifying the inhibitors have been investigated [4]. However, removing inhibitors is expensive [5] and pre-treatment methods that produce fewer inhibitors tend to yield fewer monomer sugars. Also, a detoxification phase can remove significant amounts of total fermentable sugars [5]. Thus, strains capable of robust fermentation in undiluted hydrolysates are required.

Many studies to identify genes involved in inhibitor tolerance focus on lab strains due to their ease of use [6–12]. When industrial strains are used, they are often used in combination with deletion or expression of target genes in a haploid lab strain [13–16]. For example, Van Dijk et al. used an industrial strain to identify RNA transcripts that changed with short-term adaptation to lignocellulosic inhibitors [17]. Target genes identified in the industrial strain were then separately deleted and over expressed in the lab strain BY4741. This study highlights several of the benefits of using haploid strains compared to strains of higher, and possibly unknown, ploidy. These benefits include availability of auxotrophic markers (e.g.*, leu2*∆0, *ura3*∆0, etc.) for selection and maintenance of plasmids, and ease of single copy gene deletion [18]. For adapted or evolved strains, analysis of the haploid genome is also simpler compared to analyses performed with strains of higher ploidy.

To mitigate toxicity of HMF and furfural, these inhibitory compounds are converted to less toxic alcohols by NAD(P)H-dependent oxidoreductases [19]. Some strains show an increased ability to remove furan inhibitors and previous screens with lignocellulosic hydrolysates and inhibitors such as furfural and HMF identified several *S. cerevisiae* strains with enhanced resistance [20]. As with many studies starting with natural isolates, and brewing or industrial strains, it is often challenging to validate the target gene in the same genetic background. This stems partly from the fact that many industrial strains or brewing strains do not readily sporulate and show poor spore viability [21–23]. Developing stable haploid strains from inhibitor tolerant natural isolates will facilitate identification and subsequent analysis of genes important for growth in undiluted hydrolysates.

In the work presented here, we identified a tolerant haploid strain with stable mating type that is derived from the inhibitor tolerant diploid strain YB-2625, originally isolated from bagasse. The haploid strain was then engineered for xylose fermentation by genome integration of the xylose reductase and xylitol dehydrogenase genes from *Scheffersomyces stipitis* and an additional copy of the *S. cerevisiae* xylulokinase gene. The effect of deleting *PHO13*, a loss-of-function mutation commonly identified in screens for enhanced xylose utilization, was also investigated in this genetic background. Lastly, the engineered haploid strain was compared to other commonly used haploid lab strains and showed superior inhibitor tolerance, growth on xylose, and fermentation performance in the presence of inhibitors derived from acid hydrolyzed corn stover.

## Results and discussion

### Generating and screening haploid strains for tolerance to furfural and HMF

**Fig. 1:**
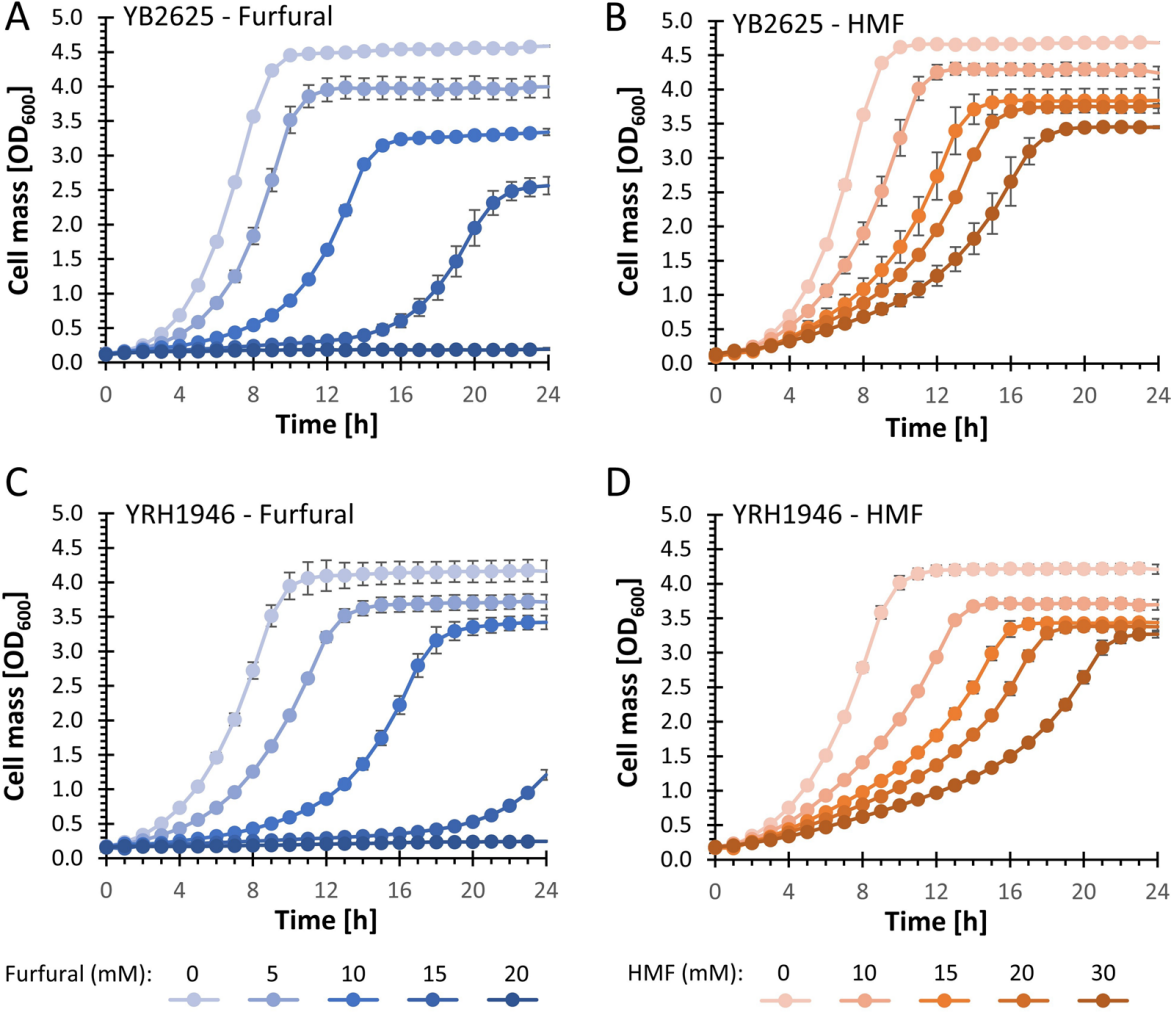
Microtiter plate growth assays with SD media in the presence of furfural (**A**, **C**) or HMF (**B**, **D**). Panels (**A**, **B**) show growth of the diploid parent strain YB-2625. Panels (**C**, **D**) show growth of the haploid strain YRH1946. Assays were performed at 30 °C with shaking every 60 s for 30 s. Error bars represent the standard deviation of a minimum of three biological replicates

In previous work comparing over 160 *Saccharomyces* strains from distilleries, breweries, and natural environments, we demonstrated that *Saccharomyces cerevisiae* strain YB-2625 showed enhanced tolerance to furfural and 5-hydroxymethylfurfural (HMF), as well as high concentrations of acid hydrolyzed corn stover [20]. In that work, the diploid strain was sequenced and shown to contain multiple genes associated with increased inhibitor tolerance. Nucleotide sequences obtained from the diploid strain revealed that the HO-endonuclease responsible for mating type switching in haploid strains [24] was homozygous and contained 11 single nucleotide polymorphisms (SNPs) and a 36 base pair deletion. Seven of the SNPs and a similar deletion in the DNA-binding domain were previously shown to render *HO* non-functional [25–27]. The lack of functional *HO* allows the formation of haploids with a stable mating type after sporulation. To identify inhibitor tolerant haploids, YB-2625 was sporulated and four 4-spore tetrads were dissected to YPD plates. Each haploid was tested for its ability to grow in the presence of furfural and HMF (data not shown). From the 16 haploids tested, four haploids (i.e.*,* one from each ascus) grew well in the presence of furfural or HMF (Additional file 1 and Additional file 2). Of the four tolerant haploid strains, three showed substantial flocculation and were not selected for further analysis. One haploid, designated strain YRH1946, did not flocculate and was further compared to its parent diploid YB-2625 for growth in the presence of furfural and HMF (Fig. 1). YRH1946 grew well in the presence of furfural or HMF but did show a slight decrease in tolerance compared to the diploid parent strain. A similar decrease in tolerance was seen with haploids derived from the diploid Brazilian industrial ethanol-producing strain PE-2 [28] and it’s been postulated that a higher surface area/volume ratio in haploid cells over diploid cells may lead to increased intracellular concentration of the inhibitor in haploids.

### Inhibitor tolerance compared to haploid laboratory strains

**Fig. 2:**
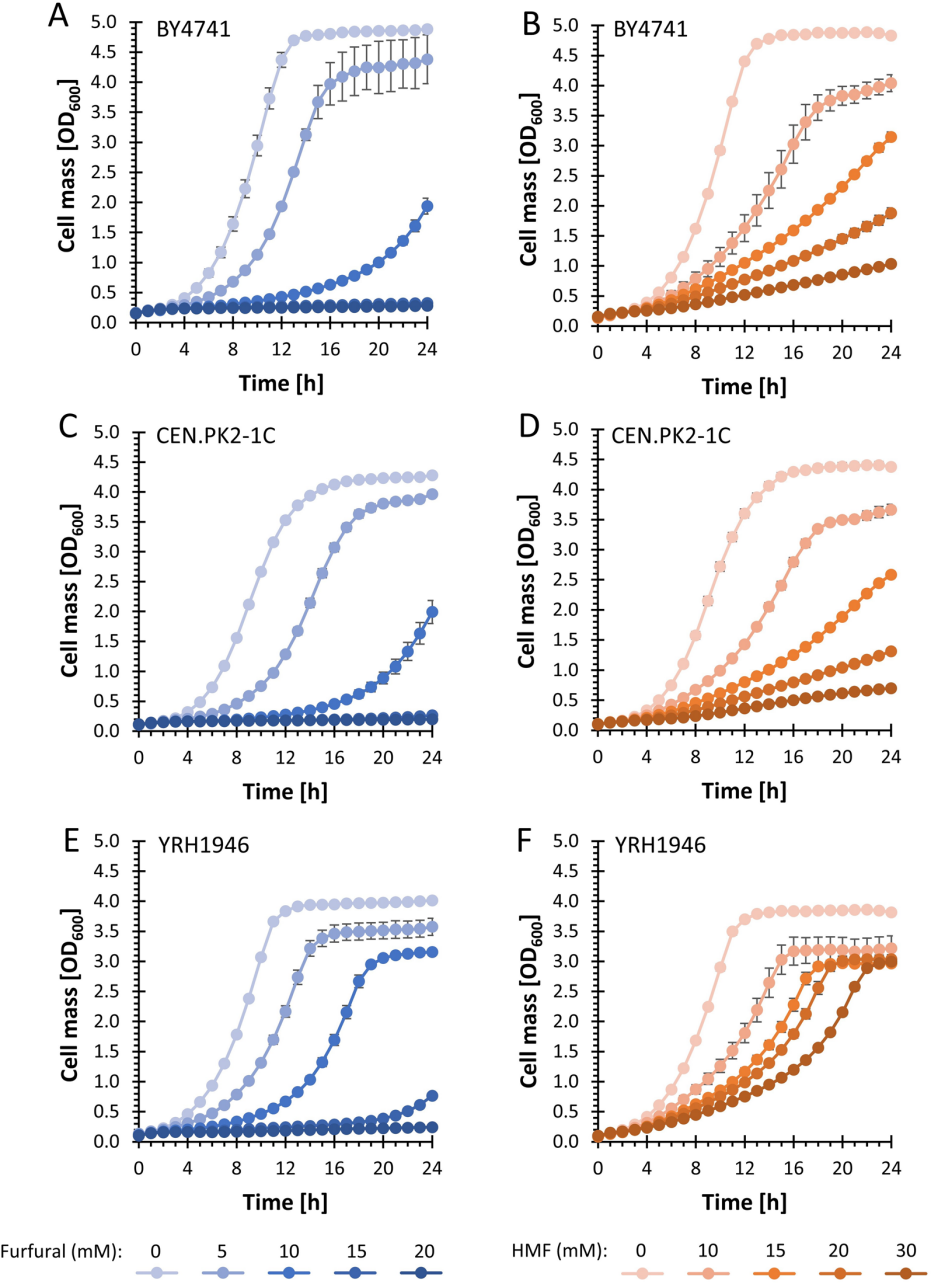
Microtiter plate growth assays with SD media in the presence of furfural (**A**, **C**, **E**) of HMF (**B**, **D**, **F**). Panels (**A**, **B**) show growth of the haploid lab strain BY4741. Panels (**C**, **D**) show growth of the haploid lab strain CEN.PK2-1C. Panels (**E**, **F**) show growth of the haploid strain YRH1946. Assays were performed at 30 °C with shaking every 60 s for 30 s. Error bars represent the standard deviation of a minimum of three biological replicates

The haploid strain YRH1946 was also compared to commonly used haploid laboratory strains BY4741 and CEN.PK2-C, with respect to growth in the presence of varying levels of furfural and HMF (Fig. 2). In the presence of furfural, YRH1946 grew better than both haploid lab strains, demonstrating shorter lag phase. With 15 mM furfural, YRH1946 started growing after 20 h, whereas the haploid lab strains were not able to grow at this concentration. None of the strains showed growth at 24 h in the presence of 20 mM furfural. YRH1946 was able to grow at the highest concentration of HMF tested while both lab strains grew poorly at HMF concentrations above 15 mM. When compared to laboratory strains, most industrial strains also show better performance when challenged with lignocellulosic inhibitors or oxidative stress [29, 30]. Furfural and HMF are known to induce oxidative stress, suggesting a role for the transcription factor *YAP1* in tolerance to these inhibitors [8, 31]. Increased expression of genes regulated by *YAP1* was seen as a common trait among six diverse *S. cerevisiae* strains analyzed in response to hydrolysate inhibitors [32]. Kim et al. 2013 [8] showed that increased expression of *YAP1* in lab strain BY4741 led to a significant increase in tolerance to furfural and HMF. That study also showed that overexpressing *YAP1* target genes for catalase (i.e., *CTT1* and *CTA1*) increases tolerance to furfural and HMF. In contrast, increased expression of the transcription factor gene *YAP1* did not lead to increased tolerance using the inhibitor tolerant YB-2625 strain [33]. Transcriptional analysis of YB-2625 compared to S288C (the parent background of strain BY4741) showed both an increase in catalase activity and expression of Yap1 regulated genes *CTT1* and *CTA1* [34] in YB-2625, indicating that mechanisms for increased tolerance are inherent in YB-2625. In this latter study, an increase in ergosterol synthesis and expression of the pentose phosphate pathway (PPP) genes *SOL1*, *GND2*, *TKL2*, and *XKS1* were also observed in YB-2625. GND (6-phosphogluconate dehydrogenase) and TKL (transketolase) activities were also previously shown to be required for tolerance to furfural [12], further indicating that increased tolerance to furfural and HMF in the YB-2625 genetic background, including the haploid derivative YRH1946, may be a direct result of natural upregulation of these activities.

### Metabolic engineering and comparison of aerobic xylose utilization

The wild type diploid bagasse isolate YB-2625, and YB-2625 engineered for xylose fermentation, were previously shown to have enhanced xylose metabolism compared to other natural *S. cerevisiae* isolates and lab strains [34, 35]. To determine if enhanced xylose metabolism was a trait that segregated with the inhibitor tolerant haploid YRH1946, the *Scheffersomyces stipitis XYL1* and *XYL2* genes for xylose reductase and xylitol dehydrogenase were integrated into the genome. An additional copy of *S. cerevisiae* xylulokinase, *XKS1*, was also integrated into the genome at the same location. For integration of genes required for xylose metabolism in YRH1946, two versions of the xylose-metabolizing haploids were constructed by targeting two different genomic regions. In strain YRH2121, integration was targeted to the *HO* gene [YRH1946 + *ho*∆::*P*_*PGK1*_-*XYL1*-*T*_*PGK1*_; *P*_*ADH1*_-*XYL2*-*T*_*ADH1*_; *P*_*HXT7*_-*XKS1*-*T*_*HXT7*_]. Integration in this region has been shown to not affect cell growth [36]. In YHR2066, integration was targeted to replace the *PHO13* gene, resulting in its deletion [YRH1946 + *pho13*∆::*P*_*PGK1*_-*XYL1*-*T*_*PGK1*_; *P*_*ADH1*_-*XYL2*-*T*_*ADH1*_; *P*_*HXT7*_-*XKS1*-*T*_*HXT7*_]. *PH013* deletion increases flux through the PPP and has been shown to increase growth on xylose [37, 38]. In a previous study with xylose-adapted lab strain CEN.PK2-1C, we also found a *pho13* loss-of-function mutation in the evolved strain that was essential for its increased in growth on xylose [39].

**Fig. 3:**
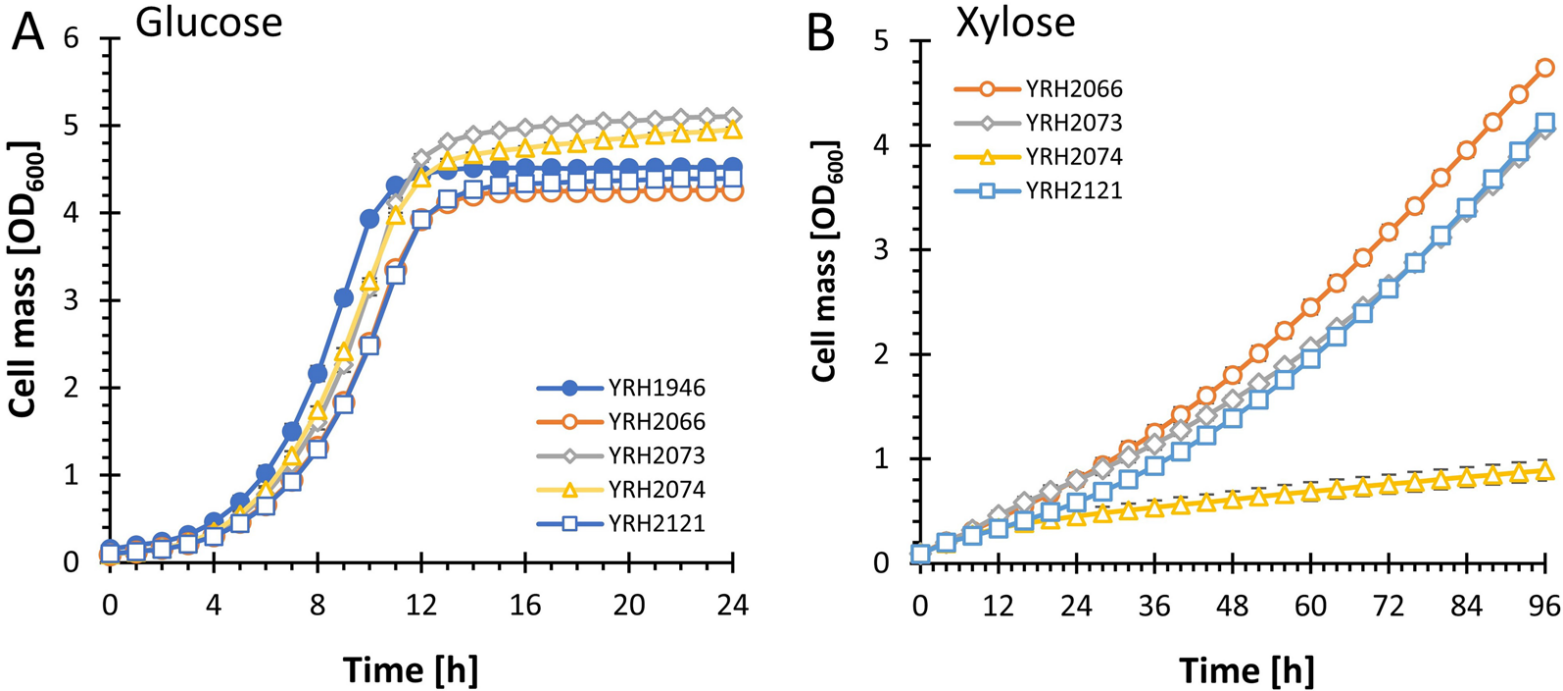
Microtiter plate growth assays with YPD (**A**) or YP5X (**B**). Assays were performed at 30 °C with shaking every 60 s for 30 s. Error bars represent the standard deviation of a minimum of three biological replicates. Strain descriptions: YRH1946 (haploid derived from YB-2625), YRH2066 (YRH1946 with genes for xylose metabolism integrated at *pho13*∆), YRH2073 (haploid lab strain BY4741 with genes for xylose metabolism integrated at *pho13*∆), YRH2074 (haploid lab strain CEN.PK2-1C with genes for xylose metabolism integrated at *pho13*∆). YRH2121 (YRH1946 with genes for xylose metabolism integrated at *ho*∆)

To generate xylose fermenting strains in haploids BY4741 and CEN.PK2-1C, the genes for xylose utilization were also targeted to replace *PHO13*. We first compared growth of the strains on glucose containing medium to ensure integration into the genome did not affect glucose metabolism or growth in general (Fig. 3A). All strains grew well when using glucose as a carbon source and no differences were observed between strains. We next analyzed growth using xylose as the only available carbon source (Fig. 3B). Deletion of *PHO13* in other genetic backgrounds results in a significant increase in growth on xylose [30, 37, 38]. Based on these previous results with deleting *PHO13* we expected to see a larger increase in growth on xylose for strain YRH2066 (*pho13*∆) compared to YRH2121 (*PHO13*). While YRH2066 with *pho13*∆ grew slightly better than strain YRH2121, in which the genes for xylose utilization were integrated at *HO*, the increase in growth was not of the order of magnitude seen in previous studies using different genetic backgrounds. One possible explanation for this result is that the strain is starting with a metabolic profile more optimized for growth on xylose compared to other strains. As mentioned above, transcriptional analysis of YB-2625 indicates that genes require for increased growth on xylose (i.e., PPP genes *SOL1*, *GND2*, *TKL2*, and *XKS1*) are already elevated [34]. Additionally, *PHO13* expression was shown to decrease ~ threefold in YB-2625 when grown on xylose/glucose mixtures [34]. This inherent reduction in *PHO13* expression in YB-2625 likely contributes to the strain’s ability to grow well on xylose containing medium when compared to other natural isolates and lab strains [35] and may explain why deletion of the *PHO13* gene results in a smaller than expected improvement in growth on xylose in this genetic background.

### Fermentation analysis in the presence of inhibitors from corn stover hydrolysate

**Fig. 4:**
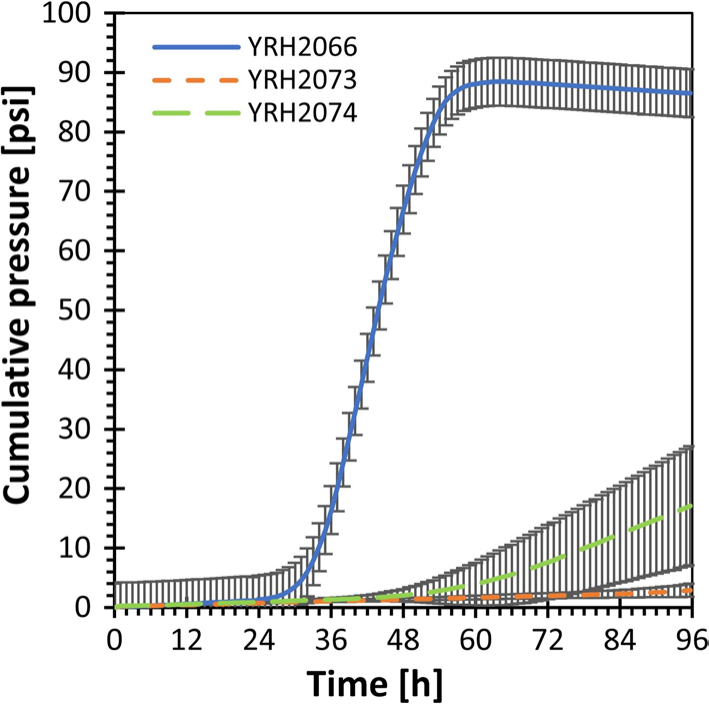
Fermentations using minimal media with corn stover hydrolysate; pH 5.0. Fermentation cultures were inoculated to an initial OD_600_ of 0.05. Fermentations were performed at 30 °C with stirring at 140 rpm. Error bars represent the standard deviation of a minimum of three biological replicates. Strain descriptions: All strains are engineered with genes for xylose metabolism integrated at *pho13*∆ YRH2066 (engineered version of YRH1946), YRH2073 (engineered version of haploid lab strain BY4741), YRH2074 (engineered version of haploid lab strain CEN.PK2-1C)

We next compared the xylose engineered haploid strains for ability to overcome inhibitors from acid hydrolyzed corn stover (Fig. 4). Fermentations were started at a low cell density in 50 mL of minimal medium supplemented with corn stover hydrolysate (CSH) for a final concentration of ~ 12 mM furfural. The CSH used for this study was prepared to generate high levels of inhibitory compounds, not for abundant monomer sugars. As such, CSH concentrations of glucose and xylose were extremely low and glucose and xylose were added to the fermentation at 40 g/L each.

Among the haploid lab strains, YRH2073, with BY4741 as the parent strain, failed to ferment even the glucose in these conditions, although furfural and HMF concentrations were reduced after 96 h (Table 1). YRH2074, with CEN.PK2-1C as the parent, ended the incubation period having lower levels of furfural and HMF than YRH2073. Consistent with the increased removal of furfural and HMF compared to YRH2073, one of the three biological replicates for YRH2074 started to ferment glucose toward the end of the fermentation, with complete removal of furfural observed (Table 1).

**Table 1:** Furfural and HMF remaining at 96 h

Strain	Parent	Furfural [g/L]	HMF [g/L]
YRH2066	YRH1946	0.00 ± 0.001	0.01 ± 0.002
YRH2073	BY4741	0.32 ± 0.095	0.33 ± 0.019
YRH2074	CEN.PK2-1C	0.00 ± 0.000	0.13 ± 0.043

Initial furfural and HMF concentrations present in the medium were 1.21 g/L and 0.44 g/L, respectively. Each parent strain listed above was engineered in the same manner to replace *PHO13* with the genes required for xylose fermentation (i.e., *pho13*∆::*P*_*PGK1*_-*XYL1*-*T*_*PGK1*_; *P*_*ADH1*_-*XYL2*-*T*_*ADH1*_; *P*_*HXT7*_-*XKS1*-*T*_*HXT7*_). Data shown are the mean values determined from at least three biological replicates ± standard deviations

Strain YRH2066 detoxified all of the furfural and HMF, and ethanol production started after a 12-h lag phase (Fig. 4). This strain also consumed all glucose present, some of the xylose, and produced 19.5 g/L of ethanol with an 80% theoretical ethanol yield based on sugars consumed (Table 2). Approximately half of the xylose consumed was directed toward producing xylitol instead of ethanol, resulting in a lower theoretical ethanol yield (80.4%) than typically seen when glucose is the only carbon source. In comparison, the engineered lab strains YRH2073 and YRH2074 showed limited sugar consumption in the presence of corn stover hydrolysate and only produced 1.1 and 4.8 g/L ethanol, respectively.

**Table 2:** Fermentation products and carbon recovery

Strain	Glucose consumed [g/L]	Xylose Consumed [g/L]	Ethanol [g/L]	Ethanol g_ethanol_/g_sugar_	Xylitol [g/L]	Carbon Recovery (%)
YRH2066	40.4 ± 0.04	7.4 ± 0.22	19.5 ± 0.15	0.41 ± 0.003	3.4 ± 0.13	96 ± 1.4
YRH2073	2.6 ± 0.59	0.3 ± 0.17	1.1 ± 0.17	0.47 ± 0.085	0.0 ± 0.08	90 ± 17
YRH2074	11.2 ± 4.31	1.3 ± 0.61	4.8 ± 2.15	0.39 ± 0.027	0.2 ± 0.10	91 ± 6.3

Fermentations were performed in batch under anaerobic conditions using corn stover hydrolysate diluted to a final concentration of 12 mM furfural (1.2 g/L). Glucose and xylose were supplemented at 40 g/L each and yeast nitrogen base was added as a nitrogen source. Data shown are the mean values determined from at least three biological replicates ± standard deviations

## Conclusions

This study sought to develop a stable haploid strain derived from an inhibitor tolerant diploid isolate, YB-2625, that was isolated from bagasse. YB-2625 was chosen for this study based on its increased ability to metabolize xylose compared to other natural isolates and lab strains as well as its superior inhibitor tolerance compared to over 160 strains isolated from breweries, distilleries, and natural environments. YB-2625 sporulates well, yielding 4-spore tetrads with high spore viability and varied degrees of inhibitor tolerance. Due to the mutations in the *HO* gene, haploids derived from YB-2625 show a stable mating type. Our results show that haploid strain YRH1946, isolated from YB-2625, maintains much of the inhibitor tolerance demonstrated by the parent strain. Compared to commonly used haploid lab strains, YRH1946 exhibited superior tolerance when grown in the presence of furfural and HMF. When engineered for xylose metabolism, this strain (YRH2066) also significantly outperformed the other haploid strains. Understanding inhibitor tolerance at a genetic level will help engineering efforts toward developing *S. cerevisiae* strains with improved growth and productivity when using biomass-derived sugars. The inhibitor tolerant, xylose fermenting, haploid strain described in this work has potential to serve as a platform strain for producing fuels and chemical from undiluted lignocellulosic hydrolysates. Further analysis of the inhibitor tolerant haploid strain’s genome, especially in comparison to non-tolerant haploids from the same genetic background, will enable identification of genes and pathways involved in tolerance to lignocellulosic hydrolysates.

## Methods

### Strains, media, and general methods

Media preparation, cell growth, transformation, and statistical analyses were performed as previously described [41]. All plasmids and microorganisms used in this study are listed in Table 3. DNA oligonucleotides used in this study are listed in Table 4. YB-2625 cells were sporulated and haploid strains were dissected from yeast tetrads on YPD plates as described in [28].

**Table 3:** Plasmids and strains

Plasmid	Description	References
*HO*-poly-*KanMX*4-*HO*	Vector for targeted integration at the *HO* locus	[36]
pRS416	pBluescript II SK + , *URA3*, *CEN6*, *ARSH4*	[42]
pRH274	pRS416 + *P*_*PGK1*_-*XYL1*-*T*_*PGK1*_; *P*_*ADH1*_-*XYL2*-*T*_*ADH1*_; *P*_*HXT7*_-*XKS1*-*T*_*HXT7*_	[43]
pRH277	*HO*-poly-*KanMX4*-*HO* + P_*PGK1*_-*XYL1*-T_*PGK1*_; P_*ADH1*_-*XYL2*-T_*ADH1*_; P_*HXT7*_-*XKS1*-T_*HXT7*_	[35]
pRH1015	PRS416 + [*pho13* homology]—*P*_*PGK1*_-*XYL1*-*T*_*PGK1*_; *P*_*ADH1*_-*XYL2*-*T*_*ADH1*_; *P*_*HXT7*_-*XKS1*-*T*_*HXT7*_—[*pho13* homology]	This study

Strains	Genotype	References
BY4741	*MAT***a** *ura3*Δ*0 leu2*Δ*0 his3*Δ*1 met15*Δ*0*	[18]
CEN.PK2-1C	*MAT***a** *ura3-52 trp1-289 leu2-3_112 his3*Δ*1 MAL2-8*^C^ *SUC2*	EUROSCARF
YB-2625	*S. cerevisiae* Diploid isolated from bagasse	ARS^a^
YRH1943	*MAT* **a** Haploid 1B isolated from YB-2625 Tetrad #1	This study
YRH1944	*MAT* alpha Haploid 2A isolated from YB-2625 Tetrad #2	This study
YRH1945	*MAT* alpha Haploid 3B isolated from YB-2625 Tetrad #3	This study
YRH1946	*MAT* alpha Haploid 4B isolated from YB-2625 Tetrad #4	This study
YRH2066	YRH1946 *pho13*∆::*P*_*PGK1*_-*XYL1*-*T*_*PGK1*_; *P*_*ADH1*_-*XYL2*-*T*_*ADH1*_; *P*_*HXT7*_-*XKS1*-*T*_*HXT7*_	This study
YRH2073	BY4741 *pho13*∆::*P*_*PGK1*_-*XYL1*-*T*_*PGK1*_; *P*_*ADH1*_-*XYL2*-*T*_*ADH1*_; *P*_*HXT7*_-*XKS1*-*T*_*HXT7*_	This study
YRH2074	CEN.PK2-1C *pho13*∆::*P*_*PGK1*_-*XYL1*-*T*_*PGK1*_; *P*_*ADH1*_-*XYL2*-*T*_*ADH1*_; *P*_*HXT7*_-*XKS1*-*T*_*HXT7*_	This study
YRH2121	YRH1946 *ho*∆::*P*_*PGK1*_-*XYL1*-*T*_*PGK1*_; *P*_*ADH1*_-*XYL2*-*T*_*ADH1*_; *P*_*HXT7*_-*XKS1*-*T*_*HXT7*_*; KanMX*	This study

^a^ Strains were obtained from the ARS Culture Collection at the National Center for Agricultural Utilization Research, Peoria, IL, USA

**Table 4:** DNA oligonucleotides

DNA oligomer #	Sequence (5′ to 3′)
7	GTGAACGTTACAGAAAAGCAG
28	GGACTAGTGTATATGAGATAGTTGATTGT
763	AAGTGGCTTGAGCTGTGGATAAGAAAAGC
764	TAATCGTCATCATTTTATTCACACCTCCGGAT
783	GAACACTTTTATTAATTCATGATCACGCTC
845	CATACCTCGCTCTGCTAATC
1029	GTTTGGCAGAGTTGGATGAATG
pho13D-F	GTTGGCCGATTCATTAATGCAGCTGGAGATACATACGTTTGTGTATACTATGCTTCTTTA TCAACTCAAGTTTTGTAGAGGAAGACGTTGAAGATGGTGATGTGACATCTTTACTATTCT CCAGCACGTTTTCAGTATTTACTTAATCGTATATTAATGACGTCCCTTATCTATTAACTT TCCGGTTTTTCTTTTTTTCGGTGAATGTTCTTTCCGTTTTAGTGAGCACGACAGGTTTCC CGACTGGAAA
pho13D-R2	GGCCTCTTCGCTATTACGCCAGCTGCAAATCATACAACTTACATAAAAACAACAAACCTG AATATTTTTCCTTTTCAAAAAGTAATTCTACCCCTAGATTTTGCATTGCTCCTCTATAAC TCATTATTGGTTAAGGTGTAGATGTCACCAAGTTTATCAATGTAAAATTTAGGTCTTGGA TAATCGTGCGAAATCTTCAAGGCTCTCTCTTCGGTTTCAATACCAGCGAAAGGGGGATGT GCTGCAAGGC

### Plasmid and strain construction

Plasmid pRH1015 was made by digesting pRH274 with *Pvu*II which cuts at sites flanking the genes for xylose metabolism. *Pvu*II digested pRH274 was then incubated with DNA fragments containing homology to direct integration to the *PHO13* gene in *S. cerevisiae* in a NEBuilder HiFi DNA assembly reaction, according to the manufacturer’s protocol (NEB). The DNA fragments were also flanked with 25 bp of homology to direct integration at the *Pvu*II sites of pRH274. DNA fragments (HiFi gBlocks) were purchased from IDT (Corvallis, IA, USA). PCR amplification of the resulting plasmid pRH1015 using primer pairs 845/28 and 7/783 confirmed that *PHO13* sequences were integrated into the plasmid. Plasmid pRH1015 was also sequenced to confirm that no mutations were generated during the cloning steps.

Yeast strains YRH1946, BY4741, and CEN.PK2-1C were transformed with *Pvu*II—linearized pRH1015 using a standard lithium acetate transformation method [44]. Cells were plated to YP5X plates to select for isolates capable of growth on xylose as a carbon source. Integration of the plasmid fragment and deletion of the *PHO13* gene in colonies growing on xylose medium was confirmed by PCR with primers 763/28 and 1029/764.

### Inhibitor tolerance and growth kinetics

Cells were grown in xylose medium using the Bioscreen C™ automated microbiology growth curve analysis system (Growth Curves USA; Piscataway, NJ, USA), which features 100 micro-well culture plates. Growth assays were performed essentially as described in [45]. Each strain was analyzed in at least quadruplicate using separate biological replicates.

### Corn stover hydrolysate (CSH) preparation

CSH was made as previously described [46] using 0.75% H_2_SO_4_, 10% solids and heating to 200 °C at 50 rpm with a 10 min hold. The CSH was adjusted to pH 5.0 using solid Ca(OH)_2_, filtered, and furfural, HMF, acetate and glucose concentrations were measured via high performance liquid chromatography (HPLC) [46]. This pretreatment method typically resulted in CSH with furfural, HMF and acetate concentrations of 52 mM, 11 mM, and 3 g/L, respectively. The CSH was not subjected to enzymatic digestion to fully release the simple sugars as we were only interested in the impacts of the inhibitors. Hydrolysate was stored at − 20 °C for later use.

### CSH Fermentation analysis

CSH fermentation analysis was performed essentially a described in [20]. Strains were cultured (50 mL) in parallel with minimal media containing CSH. Cultures were inoculated to an OD_600_ of 0.05 using cells from an overnight YPD culture. The amount of CSH used in the cultures was such that the final concentration of furfural was roughly 12 mM (1.2 g/L). The cultures were incubated at 30 °C and 140 rpm stirring. CO_2_ production was monitored using gas production measurement system (Ankom Technologies; Macedon, NY, USA). This system uses a sealed flask and measures pressure increases due to CO_2_ produced during fermentation. The system was set to measure pressure at 10 min intervals and vent when the pressure in the vessel reached 1 psi. At 96 h, samples of 1 mL each were taken to analyze residual sugars and the products formed by HPLC, following procedures reported in [45]. All experiments were performed using three biological repeats and all fermentation data calculations (i.e., yields, rates, and carbon recoveries) were performed as previously described [45].

### Statistical analyses

For experiments with three or greater biological replicates, probability analyses were performed using Student’s t-test with a two-tailed distribution and compared to the appropriate control strain. p < 0.05 was considered significant for this study. Statistical analysis was performed using Microsoft Excel.

### Supplementary Information

**Additional file 1: **Microtiter plate growth assays with SD in the presence of furfural. Panel (A) shows the diploid parent strain YB-2625. Panels (B-E) represent four most tolerant haploid progeny derived from four independent tetrads. Assays were performed at 30°C with shaking every 60 s for 30 s. Error bars represent the standard deviation of a minimum of three biological replicates.

**Additional file 2: **Microtiter plate growth assays with SD in the presence of HMF. Panel (A) shows the diploid parent strain YB-2625. Panels (B-E) represent four most tolerant haploid progeny derived from four independent tetrads. Assays were performed at 30°C with shaking every 60 s for 30 s. Error bars represent the standard deviation of a minimum of three biological replicates.

## Data Availability

The data that support the findings of this study are included within the article or the additional files.
