# Supplementary material for: Metabolic engineering of a stable haploid strain derived from lignocellulosic inhibitor tolerant Saccharomyces cerevisiae natural isolate YB-2625
Source: Biotechnol Biofuels Bioprod. 2023 Dec 6;16:190. doi: 10.1186/s13068-023-02442-9 (PMC10702107; doi:10.1186/s13068-023-02442-9)
Supplement: Supplementary file 1 — Additional file 1: Microtiter plate growth assays with SD in the presence of furfural. Panel (A) shows the diploid parent strain YB-2625. Panels (B-E) represent four most tolerant haploid progeny derived from four independent tetrads. Assays were performed at 30°C with shaking every 60 s for 30 s. Error bars represent the standard deviation of a minimum of three biological replicates. [file 13068_2023_2442_MOESM1_ESM.docx]

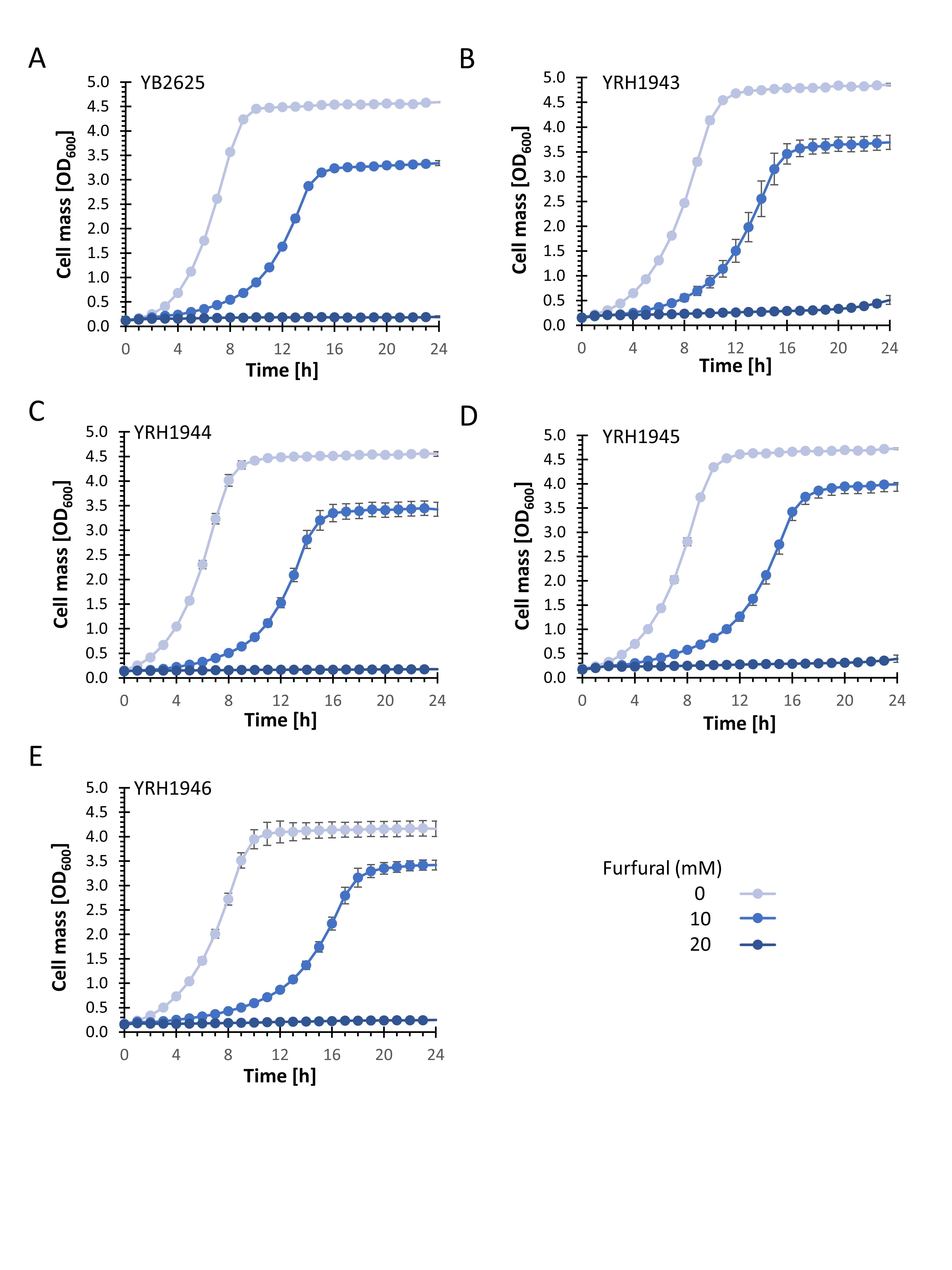


**Additional file 1. Microtiter plate growth assays with SD in the presence of furfural**. Panel (A) shows the diploid parent strain YB-2625. Panels (B-E) represent four most tolerant haploid progeny derived from four independent tetrads. Assays were performed at 30°C with shaking every 60 s for 30 s. Error bars represent the standard deviation of a minimum of three biological replicates.
